# Immunological consequences of ageing microvascular hemodynamic changes in view of cancer development and treatment

**DOI:** 10.18632/oncotarget.17749

**Published:** 2017-05-10

**Authors:** Jinhyuk Fred Chung, Sang Joon Lee, Anil K. Sood

**Affiliations:** ^1^ Xylonix Pte. Ltd., Singapore; ^2^ Division of Integrative Biosciences and Biotechnology (IBB), Pohang University of Science and Technology (POSTECH), Pohang, South Korea; ^3^ Center for Biofluid and Biomimic Research, Department of Mechanical Engineering, Pohang University of Science and Technology (POSTECH), Pohang, South Korea; ^4^ Departments of Gynecologic Oncology and Reproductive Medicine and Cancer Biology, The University of Texas, MD Anderson Cancer Center, Houston, TX, USA; ^5^ Center for RNA Interference and Non-Coding RNAs, The University of Texas, MD Anderson Cancer Center, Houston, TX, USA

**Keywords:** immunity, cancer, hemodynamics, ageing, hypertension

## Abstract

Risk factors of cardiovascular diseases have long been implicated as risk factors for carcinogenesis, but clear explanations for their association have not been presented. In this article, fundamental concepts from carcinogenesis, microvascular hemodynamics, and immunity are collectively reviewed and analyzed in context of the known features of vascular ageing effects, in formulating a theory that suggests reduced microvascular immunity as an important driving factor for carcinogenesis. Furthermore, scientific, preclinical, and clinical evidence that support this new theory are presented in an interdisciplinary manner, offering new explanations to previously unanswered factors that impact cancer risks and its treatment outcome such as chronic drug use, temperature, stress and exercise effects among others. Forward-looking topics discussing the implications of this new idea to cancer immunotherapeutics are also discussed.

## INTRODUCTION

### Limitations of current knowledge that links cardiovascular complications to carcinogenesis

**Figure 1:**
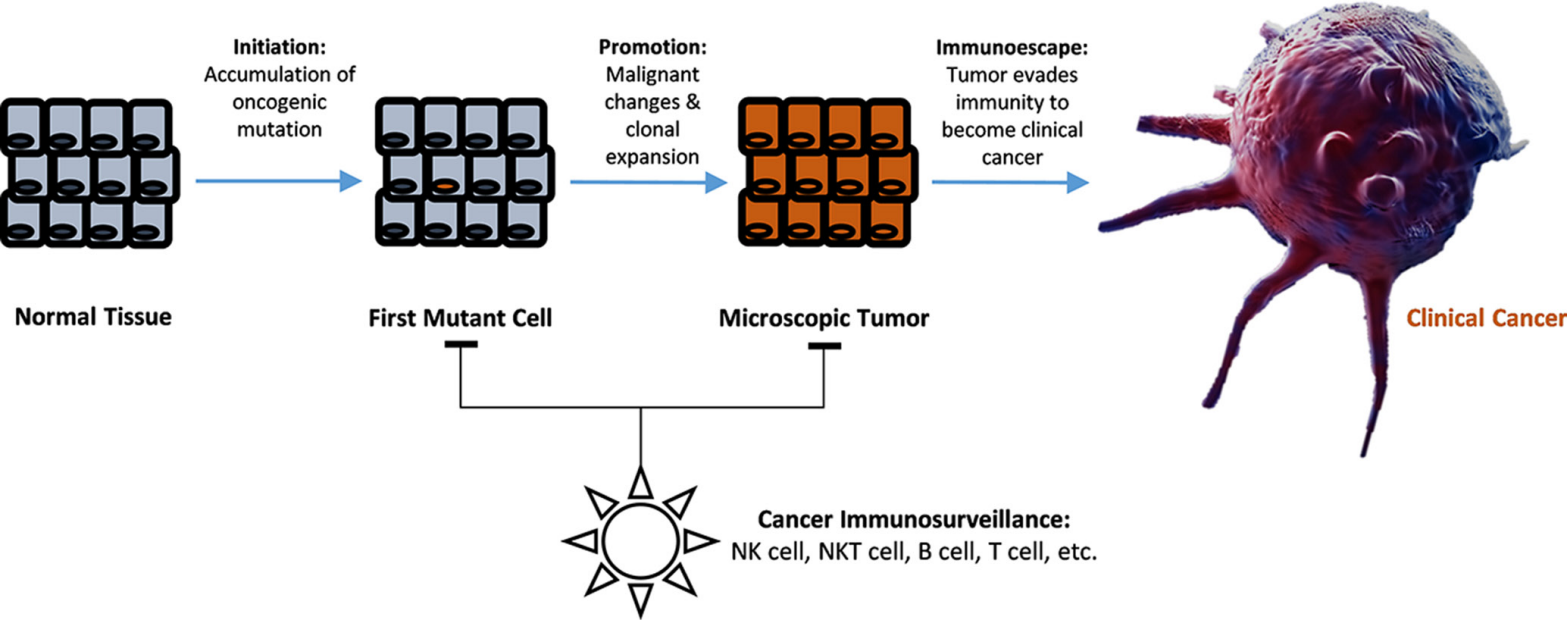
Illustrated diagram of multistage carcinogenesis model and the role of cancer immunosurveillance in preventing clinical cancer development

Cancer is a widespread and a lethal disease of neoplasms in which no reliable treatment nor its prevention methods have been developed. In explaining its natural etiology, current science interprets carcinogenesis in a largely three-step process that sequentially involves an accumulation step of oncogenic mutations, a tumor promotion step whereby the microenvironment surrounding the mutated cells fosters malignant transformation and clonal expansion of these “initiated” cells, and lastly an immuno-escape step whereby these cancerous cells overcome our tumoricidal immunity (cancer immunosurveillance) (Figure 1) [1–4]. Recently, increasing number of experimental and epidemiological evidences suggested that the epidemiological rate of carcinogenesis is mainly driven by the chronic inflammations fostering the tumor promotion step, and by the alterations in the immunity mechanism over the course of ageing, rather than by the accumulation of genetic mutations [5–7]. In context of these understandings, identification of various cardiovascular risk/preventive factors such as hypertension [8, 9], physical exercise [10], or coffee drinking [11] in association with cancer risks has been a perplexing topic as these factors seemingly have no direct contribution to any of the three steps to carcinogenesis. However, collective and in-depth understandings on hemodynamics, immunity mechanism, and the vascular ageing effects reveal a new face of cancer immunity that helps explain many of the previously unanswered controversies surrounding carcinogenesis, cancer prevention, and its treatments.

### Revisiting the fundamental basics of circulation, immunity, and the underlying mechanisms of vascular ageing

In 1951, Sternstein reported that the use of local vasodilators with antibiotics significantly improved treatment outcome of atrophic rhinitis with ozena versus the use of antibiotics alone, which was associated with increased vascularization of the nasal mucosa [12]. And more recently, Ben-Ami et al. showed that drug-resistance of *Aspergilus* fumigatus in common pulmonary infections of cancer patients was attributed to its ability to cause occlusion in pulmonary vasculatures via secretion of anti-angiogenic metabolites [13], and later showed that co-administration of pro-angiogenic factors such as basic fibroblast growth factor (bFGF) and vascular endothelial growth factor (VEGF) significantly amplified the effectiveness of antifungal treatments by increasing the accumulation of monocytes in the infected tissues [14]. Interestingly, they also reported that VEGF was less effective than bFGF in potentiating the antifungal effect due to its tendency of forming immature neovessels [14], which implicated that even the small hemodynamic changes caused by different geometric features of capillaries are critical in the effectiveness of microvascular immunity.

While hemodynamic features are relatively simple in larger vessels, blood flow in smaller ones becomes increasingly complex and heterogeneous as the dimensional constraints of capillaries (inner diameter: 5∼10 μm) approach to those of blood constituent cells (5∼15 μm) [15]. Composition and rheological properties of blood are consequently changed in the capillaries due to flow-network effects and interaction of WBCs with the endothelial lining of the draining venous vascular walls whereby the flow-rate is lower. As the ageing-associated development of endothelial dysfunctions [16–18] and rise in endogenous homocysteine (Hcy) level [19, 20] adversely influence the passage of different blood cells through capillaries by affecting both factors, pathological consequences are expected to arise.

**Figure 2:**
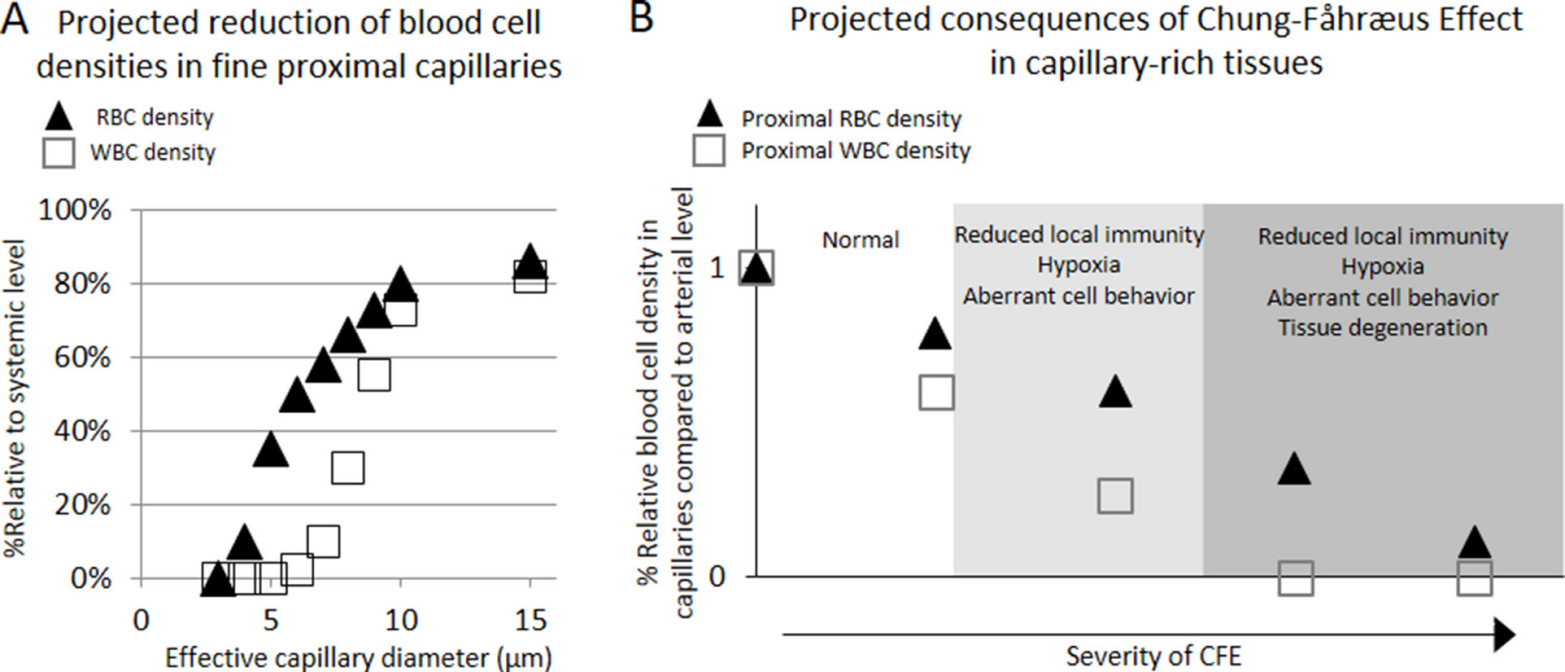
Projected density reductions in RBC and WBC flowing through proximal capillaries of different effective luminal diameters when compared to those of arterial level, and projected pathological consequences of Chung-Fåhræus Effect (CFE) (**A**) The RBC density reduction was estimated based on the published work by Tuma and Duran that described the RBC reduction phenomenon in fine capillaries per Fåhræus effect [25], adjusted by predicted volume exclusion effect against the entry of RBCs. Similarly, WBC density reduction was estimated based on the published work by Ley et al. who quantitatively showed the reduction in the density of WBC in fine mesenteric capillary networks [24], adjusted by predicted volume exclusion effect against the entry of WBCs. (**B**) Suspected pathological consequences of CFE development in capillary-rich tissues.

Briefly, phase-separation from flow-network effect arises from the fact that blood cells with different geometric configuration, viscosity, and stiffness have a general tendency of following higher velocity path with less entry restriction at each vascular bifurcation [21]. Therefore, the dimensional and network structural features of local capillary beds determine the phase-separation effect with subsequent reduction in the concentration of larger-sized blood cell components in finer capillary beds and its compensating overflow of the cells in a small number of distal channels [22]. Network Fåhræus effect is a well-known phenomenon whereby hematocrit is reduced up to 40% in the finest capillaries versus feed hematocrit [21, 23]. More importantly, Network Fåhræus effect can be extended to cause marked variations in discharge hematocrit among different capillary networks as reported by Pries et al. [21], suggesting tissue-wide variations in blood functions. Similarly, a rat model study showed marked phase-separation of WBCs in a mesenteric capillary network whereby its relative density to systemic level (6.0 × 10^9^/L) in proximal capillaries (diameter = 8.9 ± 0.4 μm) was characterized at only 55% (3.4 ± 0.5 × 10^9^/L), while the WBC density in the compensating distal capillaries (diameter = 10.1 ± 0.4 μm) was characterized at 195% (11.7 ± 2.6 × 10^9^/L) [24]. In further considerations, WBC discharge in fine capillaries less than 7 μm diameter is expected to approach “0” in an exponential fashion due to volume exclusion effects (Figure 2A). This suggests potential existence of tissue regions with hemodynamically inhibited oxygen supply and immunity cell access upon vasoconstriction in otherwise healthy individuals (Figure 2) [24, 25].

In further details, nitric oxide (NO)-modulated interaction of different WBCs with the endothelial lining of low-shear venous vascular walls and subsequent reduction of flows into capillaries is another contributing factor toward the phase-separation of blood in capillaries. Particularly, NO was shown to inhibit the activation and interaction of polymorphonuclear leukocytes (PMNs) with endothelial cells by suppressing cell stiffening and cell adhesion via CD18 expression [26], while similarly inhibiting the recruitment of monocytes via suppression of VCAM-1 gene transcription [27]. Conversely, NO plays a pivotal role in activation and recruitment of certain subsets of lymphocytes such as natural killer cells (NK cells) [28]. While delicate modulation of these interactions by NO is essential for proper immune function by the WBCs, their excessive activation is also known to cause “plugging” of capillaries that leads to significant reduction in capillary blood perfusion [29, 30]. Since the WBC plugging has a volume exclusion effect, the reduction in the effective diameter of capillaries and their draining venules would enhance the capillary flow resistance and, in turn, marked reduction of blood cell flow rate through the capillaries against the larger blood cells such as WBCs. Therefore, synergistic depression of local NK-cell activities in certain capillary beds may be expected upon reduced endothelial NO availability, owing to the increased capillary plugging effects by PMNs and monocytes [26, 27], impaired vasodilation [31–33], and suppressed NK cell activation [28].

On the basis of these hemodynamic effects, progressive exacerbation of the local blood cell reduction against larger circulating cells within and across capillary networks [21, 24] is expected due to epidemiologically characterized development of endothelial dysfunctions [18, 32] and elevation in systemic Hcy level [34] over the course of ageing. Briefly, age-dependent reduction in endothelial nitric oxide synthase and consequent reduction of NO availability in blood has been shown to cause chronic vasoconstriction from deficiencies in NO-mediated vasodilation of all types [31–33] with subsequent elevation in blood pressure that further causes progressive rarefactions in capillary beds [35, 36]. In addition, such NO-deficiency was found to reduce deformability of RBCs [37] and various WBCs [29] while aberrantly activating cell adhesion molecules (CAMs) [16, 26], further reducing the blood flow into the constricted capillaries. Meanwhile, physiologically relevant level of diet-induced hyperhomocysteinemia (10.6 ± 0.2 μM) was shown to cause increased vasoconstriction response in cynomolgus monkeys [19], and similar treatment was shown to impair NO-mediated vasodilation response by reducing eNOS level in porcine models [38]. Furthermore, the Hcy inhibition of NO-induction was shown to cause up-regulation of CAMs [20, 39]. These results implicate potential roles of age-dependent endothelial dysfunctions causing reduced endogenous NO generation [18, 32] in exacerbating the reduction of larger blood cell availability in the constricted capillary-rich tissues. For convenient discussions, we coined this exacerbating development of RBC and WBC deficiencies in capillary-rich tissues due to constrictive and/or plugging-prone endothelial dysfunctions with the term “Chung-Fåhræus effect” (CFE) [21, 24, 40].

### Projected pathological consequences of CFE

The pathological consequences upon manifestation of weakly prolonged CFE would include reduction of local immunity access, mild hypoxia, and activation of some immunity cells even at a moderate level, which may lead to pathologies arising from hypoxia-induced aberrant cell behavior, reduced regeneration, chronic infection, inflammation, and/or carcinogenesis (Figure 2B). In addition, more pronounced manifestation of the effect may further cause tissue degeneration from severe hypoxia, malnutrition, and chronic infections from local immune deficiency. Given these theoretical considerations, CFE allows for interesting interpretations on the epidemiology of known microvascular diseases including arthritis, diabetes, and Alzheimer's disease (AD) with respect to the prevalence of primary hypertension (PHT) or peripheral arterial disease (PAD) over the course of ageing. Notably, microvascular rarefaction has been implicated as the direct cause of symptomatic progression in these three diseases [41–45].

PHT constitutes about 90∼95% of all hypertension cases [46], and it arises from functional and structural capillary rarefactions causing microvascular hypoperfusion across the system with consequent increase in the systemic blood-flow resistance [47]. Briefly, the functional capillary rarefaction arises from excessive chronic vasoconstriction of arterioles, which subsequently leads to structural rarefactions of certain arterioles and capillaries that are characterized by thickened and stiffened vascular walls due to their prolonged exposure to elevated blood pressure [47]. Therefore, the prevalence of hypertension in a given age group is expected to represent the respective prevalence of constrictive and degenerative capillary disorders in the corresponding age group. PAD, on the other hand, arises from atherosclerotic narrowing of peripheral arteries to legs, arms, stomach, and head that results in reduced arterial blood-flow into the affected organs, which was shown to result in significant increase in leukocyte adhesion in venules that subsequently results in significant reduction in microvascular perfusion in the affected tissues [48]. As the atherosclerotic build-up in the peripheral arteries also increases the risks of further vascular occlusion from clot debris in the affected vascular subnetworks, PAD is expected to result in a more severe level of CFE compared to those by PHT. In short, PHT may be interpreted to represent the microvascular dysfunctions that result in weakly prolonged CFE while PAD may be interpreted to represent the microvascular dysfunctions that can potentially result in more severe level of CFE.

**Figure 3:**
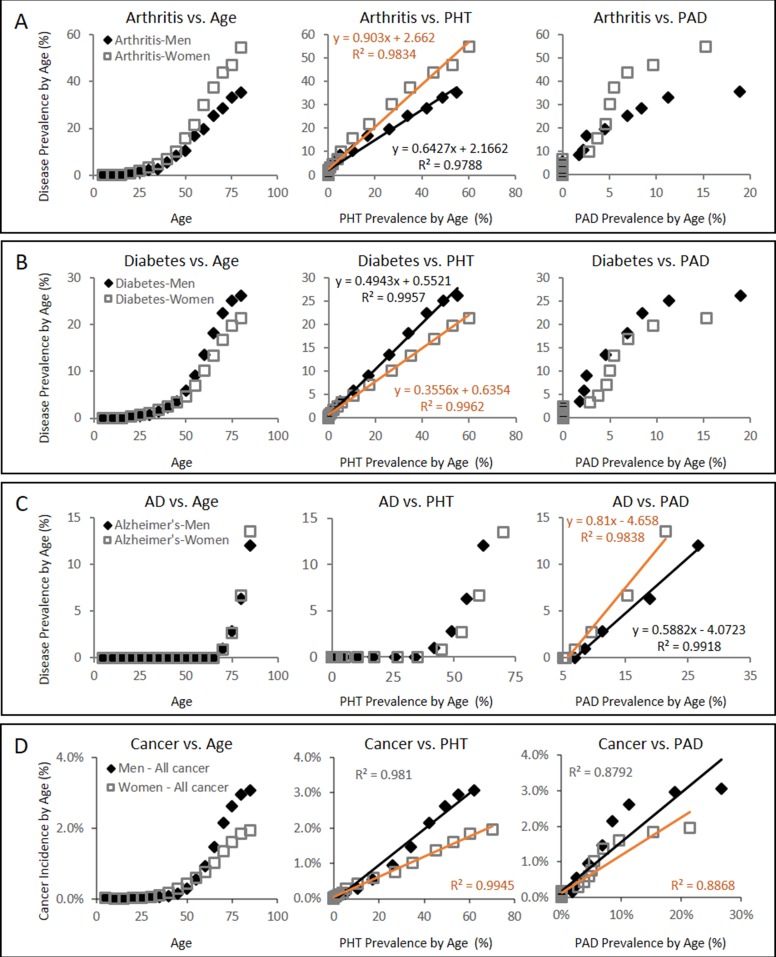
Gender specific disease prevalence (%) of arthritis, diabetes, AD, and cancer with respect to PHT or PAD prevalence (%) over the course of ageing (**A**) Arthritis prevalence vs PHT or PAD prevalence showing strong linear association between the arthritis prevalence and the PHT prevalence in both male and female groups with R^2^ values of 0.9788 and 0.9834, respectively. (**B**) Diabetes prevalence vs PHT or PAD prevalence also showing strong linear association between the diabetes prevalence and the PHT prevalence in both male and female groups with R^2^ values of 0.9957 and 0.9962, respectively. (**C**) AD prevalence, on the other hand, is characterized with strong linear association with PAD prevalence in both genders with R^2^ values of 0.9918 and 0.9838, respectively. PAD prevalence in each gender and age group was estimated from the work by Criqui and Aboyans [53]. Health statistics data on diabetes, arthritis, cancer, and hypertension were acquired from the published statistics by Public Health Agency of Canada [49–52, 54]. (**D**) Gender specific overall cancer incidence between the ages of 0 and 85 from SEER18 database of USA [100] (seer.cancer.gov, accessed on Feb 26th, 2015) with respect to PHT prevalence over the course of ageing or PAD prevalence. Their strong linear association is again observed with PHT prevalence. Due to lack of the corresponding data available in the USA, gender and age-group specific PHT prevalence data from Canada was used instead [51]. SEER18 cancer statistics registries consist of the SEER13 plus Greater California, Greater Georgia, Kentucky, Louisiana, and New Jersey, and include all cases diagnosed from year 2000 and later. It is noted that SEER18 registry excluded Louisiana cases diagnosed between July ∼ December 2005 to adjust for the impacts by Hurricanes Katrina and Rita.

Consistent with the projections regarding the pathological consequences of CFE (Figure 2B), plotting the gender and age specific prevalence of chronic microvascular diseases such as arthritis [49] and diabetes [50] against PHT prevalence [51] reveals strongly linear positive associations with correlation values (R^2^) exceeding 0.97 in both genders (Figure 3A and 3B). In turn, plotting the gender and age specific prevalence of degenerative microvascular diseases such as AD [52] against PAD prevalence [53] yet again reveals strongly linear positive associations with R^2^ exceeding 0.98 (Figure 3C).

### Evidence suggesting the involvement of endothelial dysfunction from reduced NO and CFE in clinical cancer development

**Figure 4:**
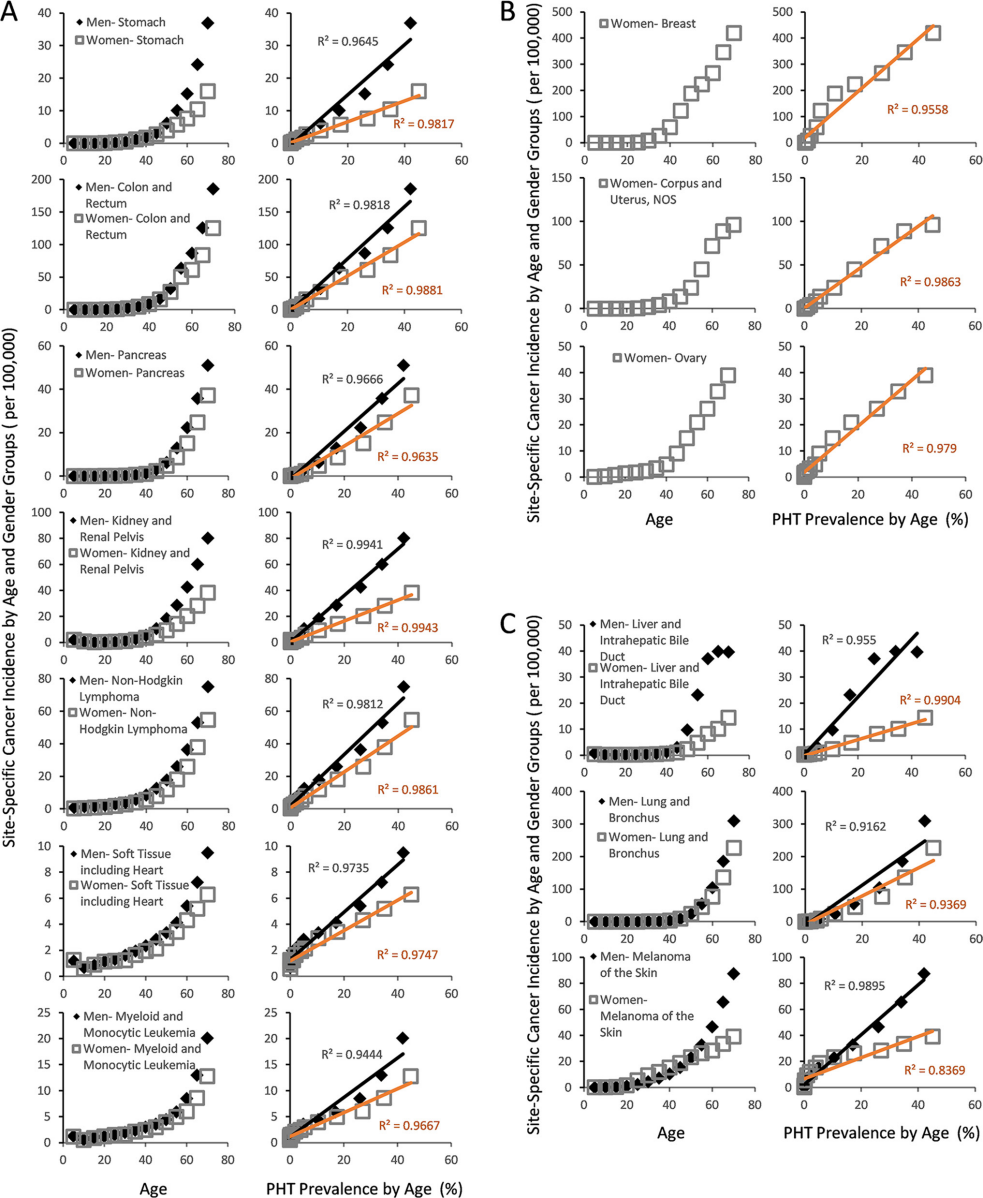
Gender and site specific cancer incidence between the ages of 0 and 70 from SEER18 database of USA [100] with respect to PHT prevalence over the course of ageing (**A**) The cancer types whose incidences are not strongly influenced by environmental exposures or genetic/gender predispositions show strongly linear association with PHT prevalence. (**B**) Female cancer types with known genetic or hormonal predispositions also show highly linear PHT prevalence, but with notable non-linear deviations. (**C**) The cancer types whose incidences are strongly influenced by environmental exposures.

**Figure 5:**
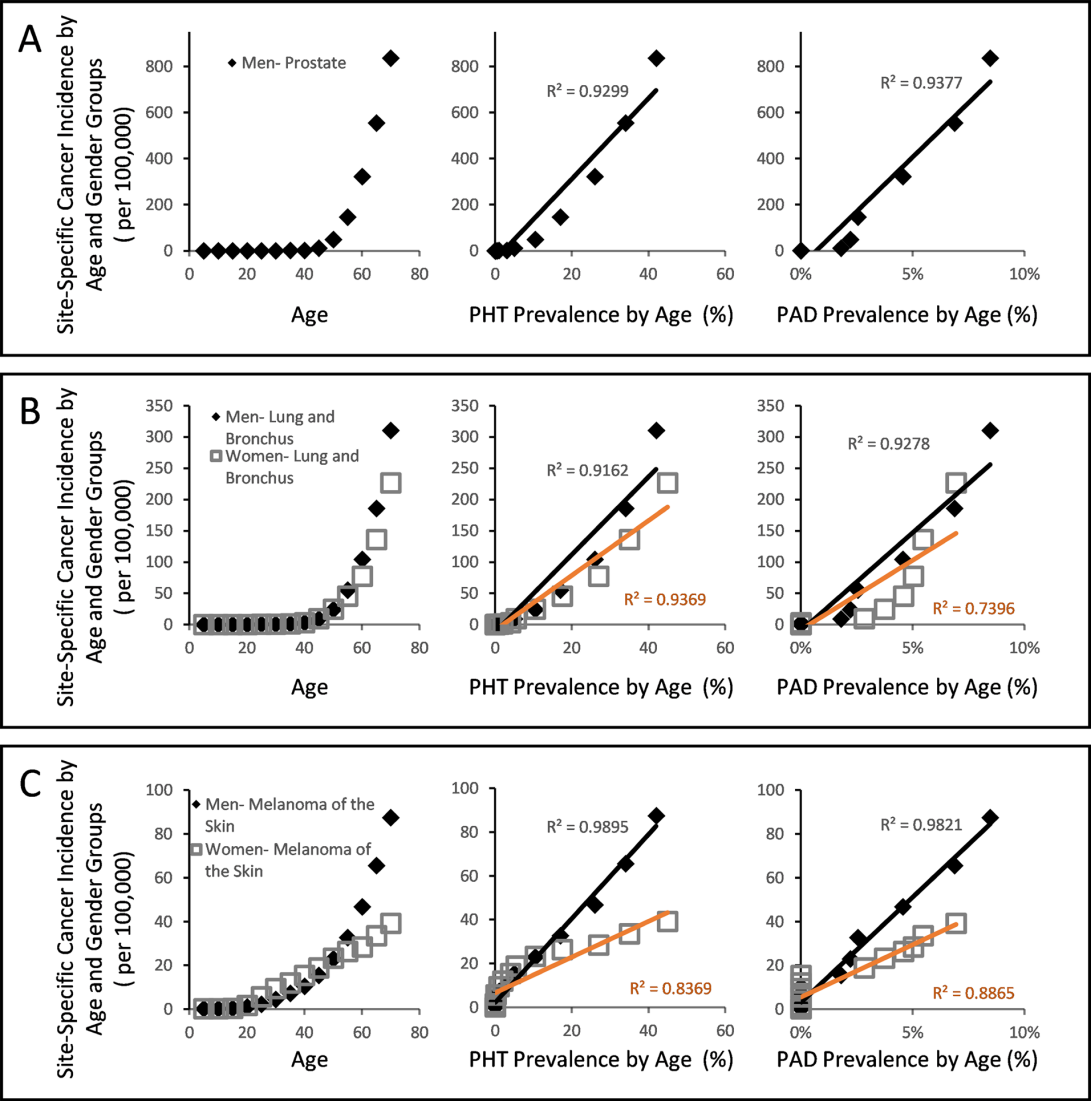
Gender and site specific cancer incidence between the ages of 0 and 70 from SEER18 database of USA with respect to PHT prevalence or PAD prevalence over the course of ageing (**A**) Male cancer of prostate incidence showed stronger linear association against PAD prevalence than against PHT prevalence. (**B**) Male cancer of lung and bronchus similarly showed stronger linear association against PAD prevalence, while the same of women did not. (**C**) Female skin melanoma showed stronger linear association against PAD prevalence than against PHT, while male skin melanoma did not.

While cancer is not yet a recognized microvascular disease, our projections raises a possibility that it may yet be another disease that arises from weak CFE (Figure 2B). Using the same approach that characterized diabetes and arthritis in strongly linear association with PHT prevalence (Figure 3A and 3B), a simple plot of overall cancer incidence in each age group from SEER18 [54] against the corresponding PHT prevalence supports this hypothesis by yielding strongly linear positive associations in both genders with R^2^ values exceeding 0.98 (Figure 3D). Application of the same analysis across 7 major cancer types without clear predisposing factors also yields strongly linear positive associations between cancer incidence of different types and PHT prevalence at R^2^ exceeding 0.96 (Figure 4A), which suggests possibilities of shared etiological factors between primary hypertension development and carcinogenesis in general. Of an interesting note, same analyses on the cancer types with known predisposing factors such as gender (Figure 4B) or environmental exposures (Figure 4C) show clearly non-linear deviations from their linear association to PHT prevalence, which well-reflects the known nature of these cancer types. Meanwhile, it is interesting to note that the incidence of certain cancer types including those of prostate, lung and bronchus of men, and skin melanoma of women are uniquely characterized in stronger linear association with PAD prevalence rather than with PHT prevalence (R^2^ of 0.9502 vs. 0.9299, 0.9278 vs. 0.9162, and 0.8865 vs. 0.8339, respectively) which suggested a somewhat different nature of these cancer development process versus other cancer types (Figure 5).

Given the well-established etiology of PHT (Figure 2A and 2B) [21, 24, 40], these observations hint that the vasoconstrictive microvascular rarefactions from dysfunctional endothelial NO production may critically contribute to clinical carcinogenesis in general via CFE. Although the observed linear associations (Figure 3, Figure 4 and Figure 5) do not qualify as evidence of this hypothesis by themselves, the hypothesis itself is alternatively supported by a store of non-clinical, retrospective and prospective studies that have shown immunity-mediated cancer preventive and antimetastatic effects of factors that promote peripheral vasodilation and endothelial NO production (Table 1). Since the initial discovery of aspirin's cancer preventive effects [55, 56], numerous retrospective studies have reported similar preventive effects across a wide range of daily drug compounds that include general non-steroidal anti-inflammatory drugs (NSAIDs) [57], metformin [58], propranolol [59], carvedilol [60], captopril [61], losartan [62], and statins [63]. And as the cancer preventive effects were compound-specific and not generalized to their drug classes in general, the shared pleotropic effects of promoting endothelial NO availability by these compounds and subsequent immunity-normalization effects were proposed as the common mechanism of their cancer preventive effects [64, 65]. In support of this idea, an NO-donating aspirin derivative (NO-aspirin) was characterized with NO-specific immunity-mediated cancer preventive effects, while the compound itself was not characterized with a direct antitumor effect [66]. Similar benefits were not observed in non-modified aspirins. And as cancer immunity is also critical in prevention of metastasis or recurrence after the initial treatments, retrospective studies have also reported that the use of incidental use of propranolol, an endothelial NO-promoting β-blocker [67], in breast cancer patients, led to significantly reduced chances of distant metastasis (*p* = 0.026) and secondary tumor formation (*p* = 0.001) via immunity-mediated effects. This led to a longer disease free interval (*p* = 0.01) and 71% reduction in breast cancer mortality after 10 years (Hazard ratio = 0.291; 95% CI = 0.119–0.715, *p* = 0.007) [68]. Prospective clinical investigation of propranolol use an as adjuvant in the pancreatic cancer treatment with Nab-paclitaxel also reported consistent results [69], further supporting its clinical benefits. Together these findings suggest that cancer immunosurveillance is critically influenced by the availability of endothelial NO, which is known to increase both microcirculation (see the review, [70]) and individual NK cell tumoricidal activity [28].

**Table 1:** Reports of cancer prognosis and treatment effect changes upon exposure to the affecters of microvascular circulation

Affecter	Study type	Effect	Cancer type	Circulation effect	Major findings
Physical exercise	human retrospective clinical study	↓ cancer specific mortality in cancer patients	colorectal [102], breast [103],	increased cardiac output and microperfusion	Dose-dependent reduction in cancer specific mortality in breast cancer patients with > 21 MET-h/wk (multivariate adjusted RR = 0.51, *p* < 0.05), in men&women with CTNNB1-negative colorectal cancer patients with > 18 MET-h/wk (multivariate adjusted RR = 0.33,*p* < 0.05).
Propranolol	human retrospective clinical study (70 propranolol users vs. 525 atenolol users)	↓ metastasis/↓ local invasiveness/↓ cancer-specific mortality risk [59]	breast	increased microperfusion from increased endothelial NO availability [67]	Use of propranolol, but not of atenolol, led to reduced cancer-specific mortality risk (HR: 0.19: 0.06–0.60), local invasiveness (OR: 0.24: 0.07–0.85), and metastasis risk (OR: 0.04–0.88).
Propranolol + Etolodac (PE)	human prospective clinical study (23 patients)	↑ progression-free survival/ ↑overall survival [69]	pancreatic	increased microperfusion [67] and anti-inflammatory effects	Combined use of PE with gemcitabine/paclitaxel (GemNab) led to increased progression-free survival (7.2 vs. 11.8 months) and overall survival (10.5 vs. 15.9 months) compared to GemNab alone.
Chemotherapy	mouse model	↑metastasis/ ↑CXCR2, CXCR4, S1P/S1PR1, PIGF and PDGF-BB in serum [104]		vasodilatory dysfunction from CXCR4 and S1P/S1PR1 overexpression. These two signals elicit vasoconstriction [105, 106].	Paclitaxel or carboplatin treatment accelerated lung metastasis with increased levels of the respective cytokines. Inhibitors of CXCR4 or S1P/S1PR1 reduced the chemo-induced metastasis and increased the median survival time by 33.9% and 40.3%, respectively.
Anti-angiogenic	mouse model	↑metastasis/ ↑tumor invasiveness [84]		vasodilatory dysfunction and capillary rarefaction [107]	Treatment with VEFGFR2 inhibitor, sutinib, or deletion of Vefg-A commonly caused increased metastasis and tumor invasiveness. This effect persisted even after cessation of anti-angiogenic treatment.
Perioperative blood transfusion	human retrospective clinical	↑tumor recurrence/ ↓survival/ ↓recurrence-free survival [108]	colon, kidney, lung, non-Hodgkin's lymphoma, etc.	microvascular vasodilatory dysfunction due to depleted NO in transfusion blood [109]	Colon cancer patients who received transfusions showed poorer survival and tumor recurrence outcome in dose-dependent manner. Similar patterns were observed in some other cancer types as well. Also, transfusion was linked with two-fold increased risk of non-Hodgkin's lymphoma. These effects are suspected to involve immunity anomalies.
Reduced housing temperature	mouse model	↑tumor progression/ ↑metastasis/ ↑carcinogenesis [110]		peripheral vasoconstriction	Housing temperature reduction from 30∼31°C to 22∼23°C nearly doubled tumor growth rate, promoted metastasis and chemical carcinogenesis in immunocompetent mice. Immunodeficient mice did not show the same effect. This effect was driven by reduced accumulation of CD8+ T cells in tumor microenvironments. Core temperature of the animals remained constant.
Surgery stress	rat model	↑lung metastasis [76]	lung	β-adrenergic activation/ ↑endothelin-1/ vasoconstriction [111]	Surgery stress reduced pulmonary marginating NK cell numbers and activity, leading to increased lung metastasis of MADB106 cancer cells by seven fold. Postoperative treatment with non-selective β-blocker nadolol and NSAID indomethacin reduced this effect by 75% (*p* < 0.0003).

### A revised view on clinical carcinogenesis and cancer treatment in context of CFE

**Figure 6:**
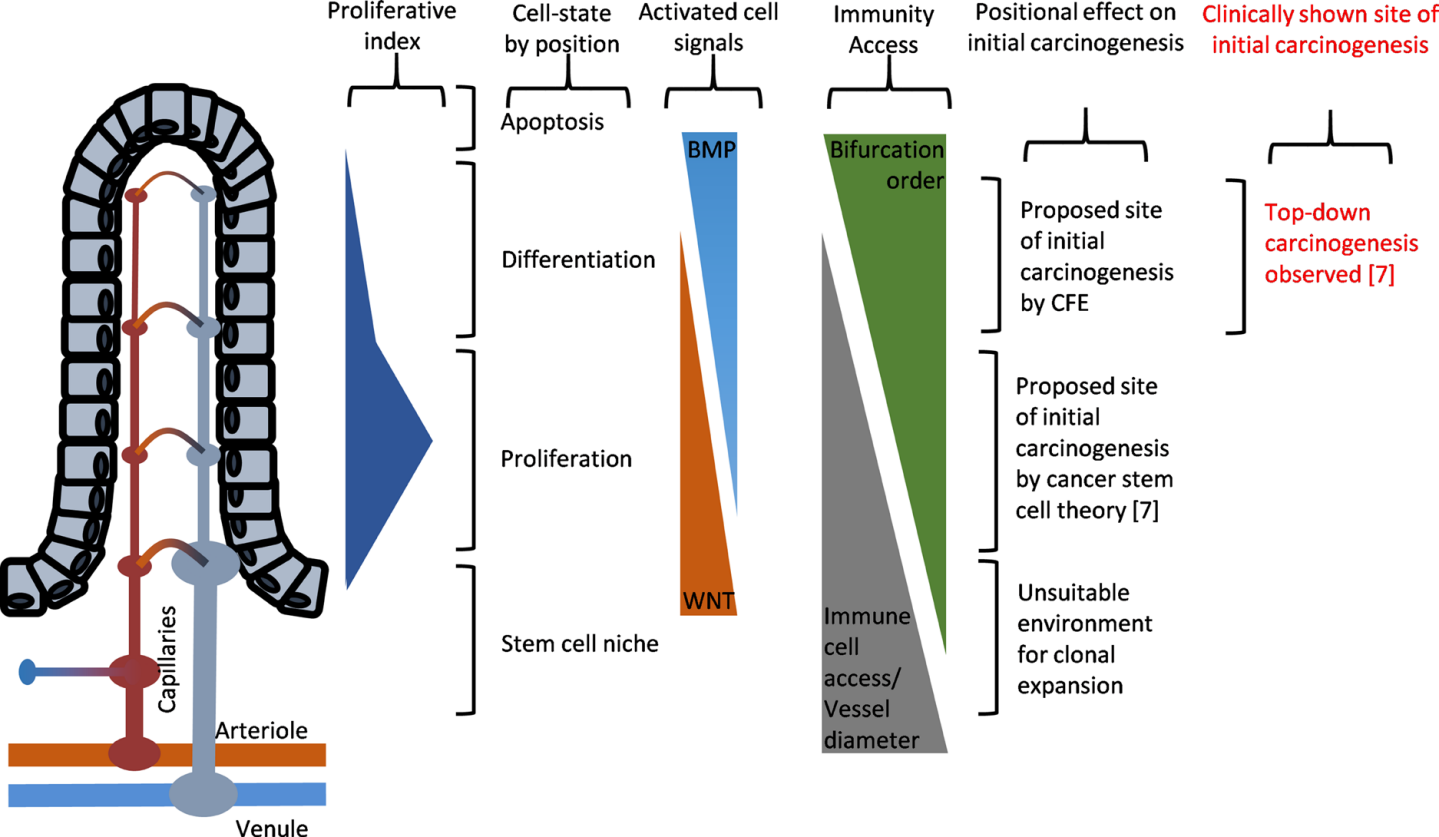
Illustrated diagram showing the paradoxical positional effects of the carcinogenesis model in the colonic crypts of colorectal cancer The proposed site of initial carcinogenesis via clonal expansion of the initiated cells, according to cancer stem cell theory, is at the bottom of the colonic crypts where the cell proliferation signals are innately up-regulated [7], but with wider capillary vessel dimensions. Evidence from clinical human specimens, on the other hand, showed that initial carcinogenesis exclusively started from the top of the colonic crypt and progressed down the colonic crypt [75]. This illustration is largely based on the description of cancer stem cell theory of colorectal cancer by McDonald *et al*. [101].

Although classical perspectives on carcinogenesis regarded age-dependent accumulation of cancer-causing mutations as the dominant causal factor in natural carcinogenesis [71], an accumulating body of evidence suggests otherwise, as oncogenic mutations were found necessary, but not sufficient, for tumorigenesis [6, 72, 73]. In explaining this paradox, it was recently suggested that the carcinogenesis in humans was likely caused by changing features of old tissues promoting cancer formation [6]. Given the fact that ageing is characterized by constrictive endothelial dysfunctions, reduced basal NO level, and progressive degeneration in capillary beds [33, 35, 36], this suggestion is consistent with the proposed theory that the progressive appearances of local tissues with hemodynamically reduced blood cell access over the course of ageing might contribute to elevating the cancer incidence by limiting the tissue access by the RBCs and, to a greater extent, immune cells. Reminiscent of tumor microenvironment theory [4], an apparent consequence of such reduced tissue access by RBCs is chronic hypoxia, which is a strong selection force and a promoting factor for carcinogenesis [74]. And as restricted immune cell access is expected in the same regions (Figure 2), increased odds of immune-escape by microscopic lesions may be expected in the ageing tissues or in those characterized with capillary rarefactions. This is a simple explanation as to how and why the tumor-promoting microenvironment emerges over the course of ageing, which no conventional theory of carcinogenesis could sufficiently explain. In this new view, carcinogenic microenvironments may be defined as suitably hypoxic capillary networks for clonal expansion within a tissue bed, with hemodynamically restricted immune cell access that allows for protected clonal expansion of the “seed” cancer cells. Consistent with our view, positional effect is observed in carcinogenesis of clinical colorectal cancer whereby carcinogenesis is exclusively started at the top end of colonic crypt where stem cell is absent and proliferation signal is paradoxically low [75]. While conventional theory of stem cell-initiated carcinogenesis in the colonic crypts of colorectal cancer cannot account for this paradox [7], our new view based on CFE simply explains such positional effect with the finest capillary structures at the top of colonic crypt whereby the immunity access is first-likely to be compromised upon onset of the ageing-related microvascular rarefaction (Figure 6).

This newly realized importance of microvascular hemodynamics in clinical carcinogenesis gives rise to the concept of effective cancer immunity (ECI), which is a natural derivative of the idea that microvascular immunity must be a composite function between the microvascular immunity cell availability and their individual cell activity (ECI = microvascular hemodynamics × individual immunity cell activity). This idea has immediate implications for optimizing current cancer treatments that are widely practiced in frontline clinics, as it implies potential links between vascular toxicity and poor microcirculation with elevated risks of distant metastasis and secondary tumor formation. In support of this idea, surgery stress with subsequent activation of β-adrenergic receptor activation alone was shown to cause reduced margination of NK cells into the lungs of rats with subsequently increased pulmonary metastatic burden, and this effect was demonstrated to be fully reversible by simple administration of a non-specific β-blocker nadolol and an NSAID indomethacin [76]. Also, cancer treatments with acknowledged vascular toxicity including radiation [77, 78], cytotoxins [79, 80], and anti-angiogenics [81] have been characterized with more frequent distant metastasis [82–86]. Although the vascular toxicities of the treatments at the primary tumor site and the subsequent hypoxia have been proposed as the primary driver of the cancer metastasis by promoting the formation of premetastatic niche in distant tissues [87], our view suggests vascular toxicities of the treatments themselves as the direct driver of spontaneous premetastatic niche and carcinogenic microenvironments formation across the system for elevated risks of metastasis and recurrence. In support of this new view, *in vivo* mouse model study showed that chemotherapeutic pretreatment with cisplatin or paclitaxel prior to tumor cell injection significantly enhanced pulmonary metastasis by increasing the endothelial cells expressing VEGFR1 [88], which was shown to be overexpressed in the endothelial cells of lung, heart, kidney, brain and liver upon systemic hypoxia [89]. Furthermore, failure of recurrent glioblastoma treatment with an antiangiogenic agent bevacizumab was reported to be primarily due to local recurrence (46%, 17/37) and formation of multifocal new enhancing lesions outside of the initial site of the disease (16%, 6/37) [90].

### CFE implications to cancer immunotherapeutics

In addition to its implications for chemoprevention, CFE may be the key in explaining the recent phase III clinical development (P3) failures of some of the most-anticipated “universal” cancer immunotherapeutics [91–93]. Briefly, late-stage clinical study results on these advanced immunity-based anti-cancer therapeutics have been disappointing despite their promising non-clinical and earlier phase clinical study results, which included, among others, a MUC1 antigen-specific Tecemotide [91] (Merck KGaA, Darmstadt), MAGE-A3 antigen-specific cancer immunotherapeutics [92] (GSK, London), and an hTERT epitope-specific peptide vaccine GV1001 [93] (Kael & Gemvax, Seoul). Given the fact that these clinical trial protocols involved adjuvant use of cytotoxin or chemoradiation therapies with severe vascular toxicities [77–79], progressive development of CFE would lead to hemodynamically inhibited microvascular immunity with effective nullification of the immunotherapeutic effects during the extended trial period of P3. In support of this view, the P3 results on Tecemotide showed that its treatment group with shorter exposure time to chemoradiation via concurrent chemoradiotherapy was characterized with longer medial overall survival (MOS) versus its placebo group (30.8 months vs. 20.6 months, respectively; adjusted HR = 0.78, 0.64–0.95, *p* = 0.016), while the treatment group with longer exposure to chemoradiation via sequential chemoradiotherapy showed no survival benefits versus its respective placebo group (19.4 months vs. 24.6 months, respectively; adjusted HR 1.12, 0.97–1.44; *p* = 0.38). In further support of the adverse role of CFE and vascular toxicity against effective cancer immunity, the phase III clinical trial study on GV1001 showed successful induction of immune response without clinical efficacy [93]. Particularly, its post-trial analysis reported paradoxically enhanced survival benefit among pancreatic cancer patients with elevated level of the pro-inflammatory chemokine eotaxin (14.8 [10.1–20.5] months MOS in high eotaxin group versus 7.9 [5.9–11.3] months MOS in low eotaxin group; *p* = 0.0135) [94], which is known to elicit angiogenic responses *in vivo* [95] and a significant increase in endothelial NO production [96]. More importantly, it was also reported that only the patients with preserved high eotaxin level during the chemoimmunotherapy regimen were characterized with longer MOS, while those whose eotaxin level was reduced by the chemoimmunotherapy regimen did not.

Together, our CFE-based interpretation of the P3 findings from these advanced immunity-based cancer therapeutics suggest that similar cancer-targeting immunotherapeutics may be incompatible with conventional cytotoxins and chemoradiation treatments. Conversely, these considerations raise a possibility that their efficacies may be optimized by incorporating endothelial NO-inducing or NO-donating drugs such as carvedilol [97], nebivolol [97], statins [98], or metformin [99].

In a concluding remark, future prospective clinical investigations are needed in optimizing the current cancer treatment regimens via minimization of their inherent vascular toxicities. As there are many approved vasoprotective drug agents of historical safety and economic affordability with demonstrated cancer preventive and antimetastatic effects [64], confirmation of their adjuvant cancer treatment benefits may allow for an highly effective and affordable improvements in cancer treatment at low economic costs. Also, the theoretical considerations surrounding CFE raise the possibilities of future research venue involving biophysical data of microvascular health and its use in pre-onset risk assessment of cancer metastasis, recurrence, and perhaps the very first carcinogenesis itself. Incorporation of the enabling information technologies into clinical cancer research will be vital for advancing such developments.
